# Supplementary material for: N58A Exerts Analgesic Effect on Trigeminal Neuralgia by Regulating the MAPK Pathway and Tetrodotoxin-Resistant Sodium Channel
Source: Toxins (Basel). 2021 May 17;13(5):357. doi: 10.3390/toxins13050357 (PMC8157219; doi:10.3390/toxins13050357)
Supplement: Supplementary file 1 [file toxins-13-00357-s001.zip › toxins-1186420-supplementary.pdf]

Supplementary Materials: N58A Exerts Analgesic Effect on Trigeminal Neuralgia by Regulating MAPKs Pathway and Tetrodotoxin-Resistant Sodium Channel

Chun-Li Li, Ran Yang, Yang Sun, Yuan Feng, Yong-Bo Song

S1. Sequence Alignment of Syb-prII and the Structure Prediction

The sequence alignment of Syb, Syb-N58A, LqhIT2, BmKIM, BmKITa, BmKITb, and BmKANEP3 is shown in Figure S1-1. By predicting the secondary structure, the position and coverage of the β-fold and α-helix of the target sequence and the template sequence are basically the same. The above results indicate that Syb and Syb-N58A likely belong to the β-anti-excitatory neurotoxin. The crystal structure of LqhIT2，one of the depressant anti-insect neurotoxins, has been resolved (PDB ID: 2I61). The sequence identity of Syb and LqhIT2 was 83.61% and the sequence identity of Syb-N58A and LqhIT2 was 81.97%. Therefore, 2I61 was selected as modeling template for Syb and Syb-N58A.

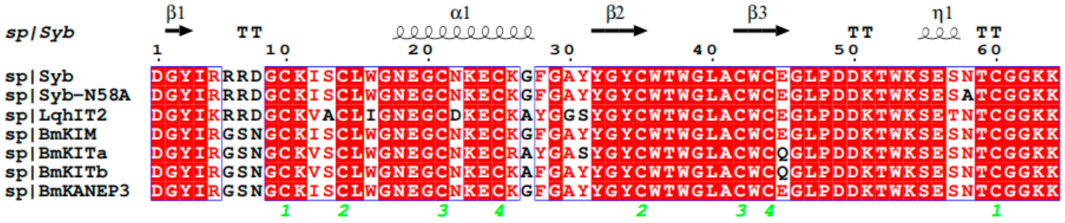

Figure S1-1. The sequence alignment between Syb, Syb-N58A, LqhIT2, BmKIM, BmKITa, BmKITb, and BmKANEP3.

Modeller 9.9 [1] was used to build 500 conformations of Syb and Syb-N58A following sequence alignment with ClustalX 2.1 [2]. The models with the highest discrete optimized protein energy scores were validated by Ramachandran plots and the best performer was selected. The amino acid residue of Syb in the optimal conformation was 93.8% in the optimum region and 6.2% in the allowable region. The amino acid residue of Syb-N58A in the optimal conformation was 89.8% in the optimum region and 10.2% in the allowable region. It can be seen that the proportion of amino acid residues in the maximum allowable region exceeded 90%, and the conformation of the model was considered to be in accordance with the rules of stereochemistry.

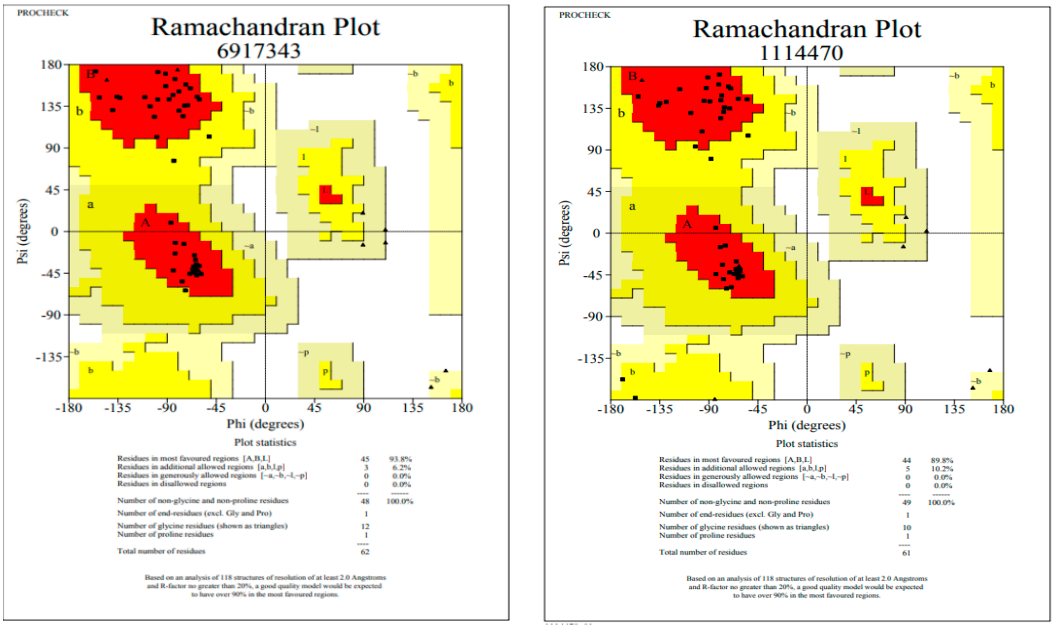

(**a**) (**b**)

Figure S1-2. The Ramachandran plot of Syb (**a**) and Syb-N58A (**b**).

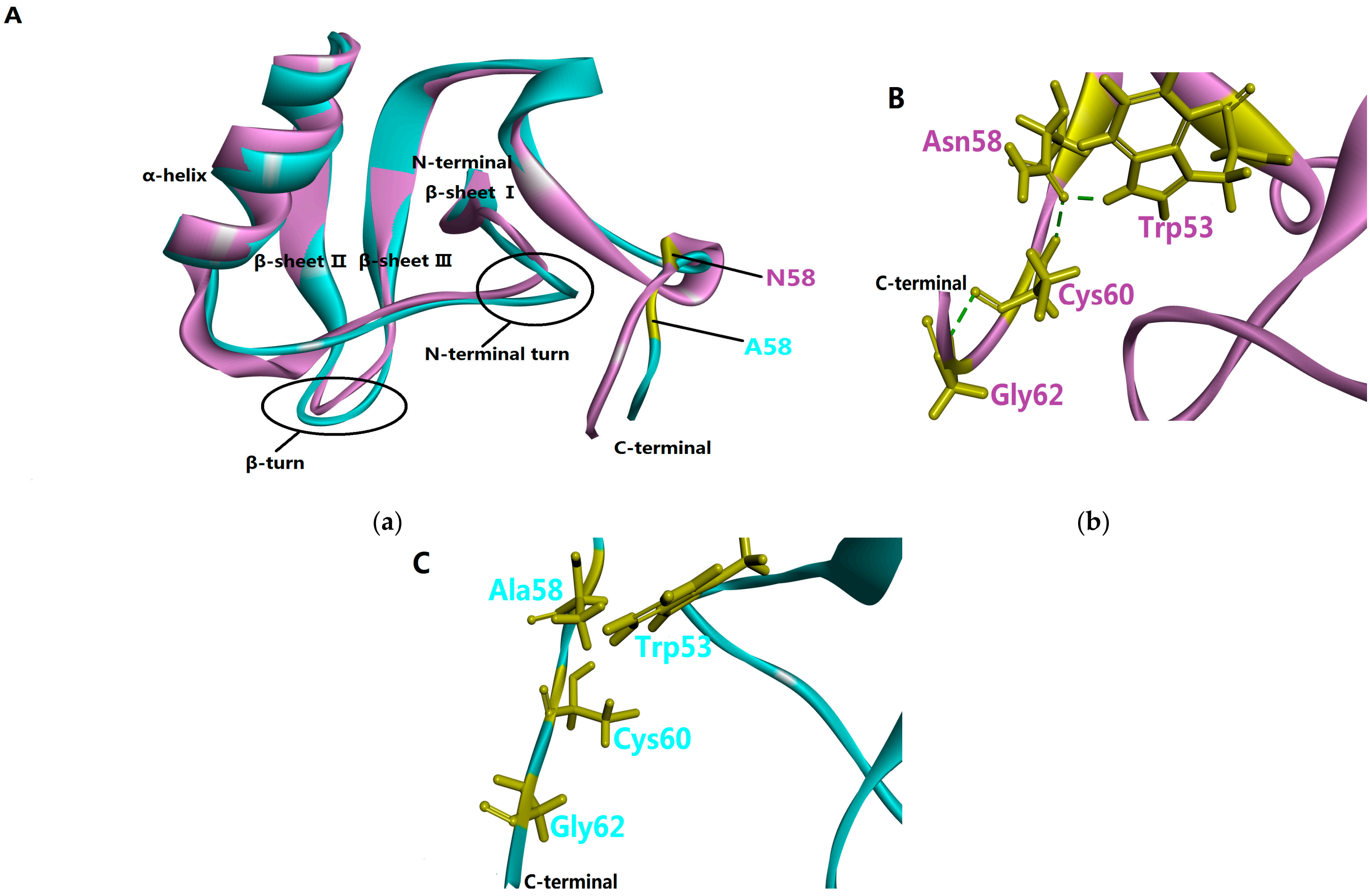

(**a**) (**b**)

(**c**)

Figure S1-3. Superimposed structures of Syb (purple) and Syb-N58A (light blue). (**a**) the interactions of N58 in the C-terminal of Syb. (**b**) The interactions of A58 in the C-terminal of Syb-N58A. (**c**) Amino acid residues were labeled in glassy yellow. H-bonds were labeled in green.

The double-precision MD simulation method of Amber18 was used to simulate the statuses of the homology modeling structures of Syb and Syb-N58A in a real physiological environment. After MD simulation, the best structures were selected to perform further analysis. The molecular overlay of Syb and Syb-N58A is shown in Figure S1-3(A). The backbone of the two structures had a high degree of similarity, but two regions (β-turn and N-terminal turn), which were highly conserved in all of the β-anti-excitatory neurotoxin [3], were observed shifting downward after mutation. In the structure of Syb, there are three intramolecular H-bonds between Trp53, Asn58, Cys60, and Gly62 in the C-terminal. Compared with the wild type, these intramolecular H-bonds of Syb-N58A were disrupted. Those changes may be responsible for its activity and affinity for the target [4].

Previous studies showed that the unique structure of N58 plays a vital role in toxin activity [5]. The results of the mutation experiment with N58A showed a significant decrease in binding affinity, possibly due to the fact that the amide side chain of Asn, which can act as a hydrogen bond donor, strongly interacts with Trp53 and Cys60 [4], which is also consistent with our results.

S2. Induction Expression and Soluble Analysis of Expression Vectors pSYPU-3c-*Syb-prII*

The rBmK syb-N58A was expressed using the pSYPU-2c vector in the *E. coli* BL21 (λDE3) and mostly in soluble form, and then was isolated from soluble fractions in the *E. coli* BL21 (λDE3) cell lysate and purified to homogeneity by nickel affinity chromatography followed by 15 % SDS-PAGE. Syb-N58A was finally eluted with buffer C (Figure S2-1A) and the size was about 9kDa (Figure S2-1b, line C). Analysis of the SDS-PAGE revealed that the culture supernatants contained predominantly the soluble recombinant peptide. Purity was greater than 90% as judged by Coomassie staining of the SDS-PAGE gel (Figure S2-1B).

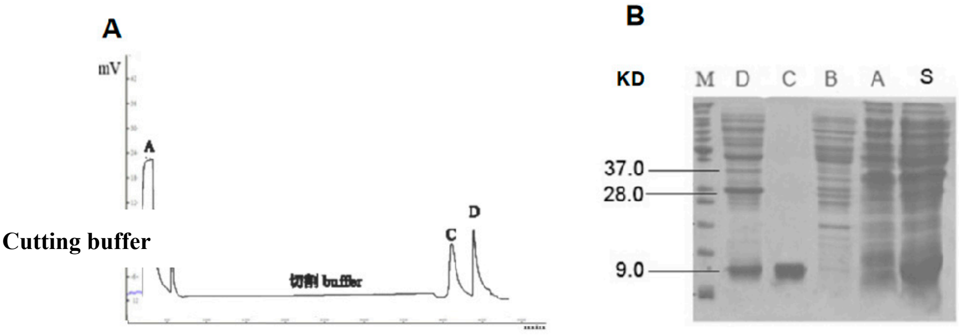

Figure S2. Purification of SYPU-N58A by Chelating Sepharose Fast Flow. (**A**) The metal chelating affinity chromatography; (**B**) 12.5% SDS-PAGE analysis.

References

1. Webb B, Sali A. Comparative Protein Structure Modeling Using MODELLER. *Current protocols in protein science.* **2016,** 86, 2.9.1-2.9.37.
2. Kohli D, Bachhawat A. CLOURE: Clustal Output Reformatter, a program for reformatting ClustalX/ClustalW outputs for SNP analysis and molecular systematics. *Nucleic acids research.* **2003**, 31, 3501-3502.
3. Zhang, Joel Z, Yarov-Yarovoy, et al. Structure-Function Map of the Receptor Site for β-Scorpion Toxins in Domain II of Voltage-gated Sodium Channels. *Journal of Biological Chemistry.* **2011,** 286, 33641-33651.
4. Karbat I, Turkov M, Cohen L, et al. X-ray Structure and Mutagenesis of the Scorpion Depressant Toxin LqhIT2 Reveals Key Determinants Crucial for Activity and Anti-Insect Selectivity. *Journal of Molecular Biology.* **2007**, 366, 586-601.
5. Saucedo, A. L, Rio-Portilla D, et al. Solution structure of native and recombinant expressed toxin CssII from the venom of the scorpion Centruroides suffusus suffusus, and their effects on Nav1.5 Sodium channels. *Biochim Biophys Acta*, **2012**, 1824, 478-87.
